# Prognostic factors associated with early recurrence following liver resection for colorectal liver metastases: a systematic review and meta-analysis

**DOI:** 10.1186/s12885-024-12162-4

**Published:** 2024-04-08

**Authors:** Yuan Tian, Yaoqun Wang, Ningyuan Wen, Shaofeng Wang, Bei Li, Geng Liu

**Affiliations:** 1 Department of General Surgery, Division of Biliary Surgery, West China Hospital, Sichuan University, Sichuan, Chengdu, 610041 China; 2 Research Center for Biliary Diseases, West China Hospital, Sichuan University, Sichuan, Chengdu, 610041 China

**Keywords:** Colorectal liver metastases, Early recurrence, Prognostic factors, Liver resection, Meta-analysis

## Abstract

**Background:**

Colorectal cancer (CRC) is the 3rd most common malignancy with the liver being the most common site of metastases. The recurrence rate of colorectal liver metastases (CRLM) after liver resection (LR) is notably high, with an estimated 40% of patients experiencing recurrence within 6 months. In this context, we conducted a meta-analysis to synthesize and evaluate the reliability of evidence pertaining to prognostic factors associated with early recurrence (ER) in CRLM following LR.

**Methods:**

Systematic searches were conducted from the inception of databases to July 14, 2023, to identify studies reporting prognostic factors associated with ER. The Quality in Prognostic Factor Studies (QUIPS) tool was employed to assess risk-of-bias for included studies. Meta-analysis was then performed on these prognostic factors, summarized by forest plots. The grading of evidence was based on sample size, heterogeneity, and Egger’s *P* value.

**Results:**

The study included 24 investigations, comprising 12705 individuals, during an accrual period that extended from 2007 to 2023. In the evaluation of risk-of-bias, 22 studies were rated as low/moderate risk, while two studies were excluded because of high risk. Most of the studies used a postoperative interval of 6 months to define ER, with 30.2% (95% confidence interval [CI], 24.1–36.4%) of the patients experiencing ER following LR. 21 studies were pooled for meta-analysis. High-quality evidence showed that poor differentiation of CRC, larger and bilobar-distributed liver metastases, major hepatectomy, positive surgical margins, and postoperative complications were associated with an elevated risk of ER. Additionally, moderate-quality evidence suggested that elevated levels of carcinoembryonic antigen (CEA) and carbohydrate antigen 19–9 (CA199), lymph node metastases (LNM) of CRC, and a higher number of liver metastases were risk factors for ER.

**Conclusion:**

This review has the potential to enhance the efficacy of surveillance strategies, refine prognostic assessments, and guide judicious treatment decisions for CRLM patients with high risk of ER. Additionally, it is essential to undertake well-designed prospective investigations to examine additional prognostic factors and develop salvage therapeutic approaches for ER of CRLM.

**Supplementary Information:**

The online version contains supplementary material available at 10.1186/s12885-024-12162-4.

## Introduction

Colorectal cancer (CRC) is the 3rd most common malignancy and the 2nd most deadly cancer worldwide [1]. It is highly prevalent in developed countries but has started to show an increasing trend in China, partially attributed to shifts toward a high-fat, low-fiber diet [2]. CRC is prone to distant metastases, affecting over 50% of patients, with the liver being the primary site in approximately 70% of cases [3, 4]. Therapeutic options for colorectal liver metastases (CRLM) include hepatectomy, chemotherapy, radiotherapy, hepatic artery embolization, and thermal ablation, such as microwave coagulation therapy, radiofrequency ablation [5]. Currently, liver resection (LR) is acknowledged as the most effective treatment for CRLM patients, which can offer prolonged survival and, in selected cases, a chance of cure [6]. Increasing effectiveness of chemotherapy regimens, advances in surgical techniques, and improvements in perioperative patient management have expanded the boundaries of resectability [7, 8]. The current consensus proposes that a disease should be considered technically resectable as long as complete macroscopic resection is feasible while maintaining at least a 30% future liver remnant [9, 10]. Nevertheless, not all technically resectable patients experience a survival benefit from surgery, with 3-year recurrence rates reaching 60–70% [11–13]. The earlier the recurrence, the worse the prognosis, but the definition of early recurrence(ER) varies from 6 to 24 months [14–16].

Therefore, this meta-analysis aims to elucidate prognostic factors associated with ER in CRLM patients undergoing LR. Subsequently, our objective is to identify individuals with high risk of ER, who might benefit from closer surveillance and appropriate salvage therapy.

## Materials and methods

### Protocol and reporting

The protocol for this study was registered on PROSPERO (International Prospective Register of Systematic Reviews, www.crd.york.ac.uk/prospero) with the registration number CRD42023444091. This review was carried out according to the Preferred Reporting Items for Systematic Reviews and Meta-analyses (PRISMA) guidelines [17]. The PRISMA checklist is available in Supplementary Table S1.

### Data sources and search strategy

All potentially eligible publications were retrieved from PubMed, Embase, Cochrane, and Web of Science from database inception until July 14, 2023. The search, employing the keywords “colorectal liver metastases”, “surgery”, “early”, and “recurrence”, was carried out by two investigators (YT, SFW). Supplementary File 1 included detailed information on the search strategy. Additionally, the bibliographies of included articles and relevant reviews were manually scrutinized to identify additional research and explore potentially relevant studies.

### Eligibility criteria and study selection

Subjects were eligible for inclusion if the following criteria were met: (1) Prospective or retrospective studies including patients with CRLM who received liver resection; (2) Articles presenting ER rates categorized by a prognostic factor; (3) Articles reporting a relative ratio (RR) or an odds ratio (OR) (with a 95% confidence interval [CI]) or offering adequate data for RR/OR estimation; (4) No language restrictions.

Studies were excluded according to the following criteria: (1) Articles on palliative surgery; (2) Articles without sufficient data for analysis; (3) Experimental animal studies; (4) Reviews, commentaries, conference proceedings, letters, case reports, editorials, and meta-analysis.

Article screening and study selection were independently performed by two reviewers (SFW, YQW). In instances of discordance, resolution was achieved through collaborative deliberation within the research team, culminating in a final consensus.

### Data extraction

The following data were extracted from each included study, and missing data were noted: (1) first author, publication year, country, period of recruitment, study type, patient count, follow-up period, overall recurrence rate, ER definition, ER rate, 5-year OS in the ER group, and inclusion/exclusion criteria (Table 1); (2) Prognostic factors, including patient characteristics (continuous variables: age, carcinoembryonic antigen [CEA], carbohydrate antigen 19–9 [CA199], binary variables: gender), primary tumor characteristics (binary variables: tumor differentiation [poor vs moderate/good], lymph node metastases [LNM], tumor stage [T3-4 vs T1-2], tumor location [rectum vs colon]), liver metastases characteristics (binary variables: number [more vs less], diameter [> 5 cm vs ≤ 5 cm], synchronous metastases, bilobar distribution, extrahepatic metastases), and therapeutic factors (binary variables: laparoscopic resection,simultaneous resection, major hepatectomy,surgical margins [positive vs negative],preoperative chemotherapy, postoperative chemotherapy,blood transfusion,postoperative complications) and clinical risk score [CRS, binary, > 2 vs ≤ 2]; (3) RRs or ORs and corresponding 95% CIs for association between each prognostic factor and ER.

**Table 1:** Included study characteristics

First author (year, country)	Study period	Study design	No. of patients	Median follow-up (months)	ER definition (months)	Overall recurrence rate (%)	ER rate (%)	5-year OS in ER group (%)	Inclusion criteria	Exclusion criteria
Bhogal,2015 [18],UK	2004–2006	PC	243	58	18	52.7	38.3	-	LR for CRLM	-
Chen,2022 [19], China	2008–2020	RC	144	-	11	-	-	-	Histologic CRLM, LR after NAC;	NAR, lack of f/p data
Dai,2021 [15], China	2012–2019	RC	202	-	6	77.7	43.6	-	Synchronous CRLM, adenocarcinoma, curative-intent surgery	recurrent CRLM or remnant lesions, lack of f/p data
Deng,2023 [20], China	2008–2020	RC	323	-	13	-	-	-	Clinical or histological CRLM; simultaneous curative-intent resection	Lack of f/p data, other severe diseases
Finkelstein, 2008 [21],USA	1995–2002	PC	100	31	12	52	30.0	-	LR for CRLM	Extrahepatic disease
Imai,2016 [22],France	1990–2012	PC	846	57.6	8	78.8	44.8	11.1	Curative surgery for CRLM, a f/u > 2 years	Died of postoperative complications
Inoue,2020 [23], Japan	2001–2017	RC	295	-	6	64.1	29.8	45	LR for CRLM	Noncurative resection
Jung,2016 [24], Korea	1990–2011	RC	277	45.1	6	-	10.8	33.8	LR for CRLM	R2 resection
Kaibori,2012 [14], Japan	1993–2007	RC	119	31	24	53.8	45.4	24.2	Curative resection for CRLM	R2 resection
Lalmahomed,2015 [25], Netherlands	2008–2012	RC	151	28	12	76.2	54.3	-	Adult, adenocarcinoma, CRLM, LR or open RFA	Noncurative resection, extrahepatic metastasis, lack of f/p data
Lin,2018 [26], China	1999–2016	RC	307	31.7	6	57.3	16.0	-	CRLOM, adenocarcinoma, R0 resection, a f/u > 6 months	Extrahepatic metastasis, R1 or R2 resection, loss to f/p
Liu,2015 [27], China	2000–2014	RC	303	40	12	63.4	47.9	16	LR for CRLM	-
Malik,2007 [28], UK	1993–2003	PC	430	33	6	66.7	20.0	22	LR for CRLM, a f/u > 2 years	NAC, repeated LR
Mao,2017 [29],China	2007–2015	PC	255	28.6	6	65.1	34.1	11.8	Curative-intent LR for histological CRLM, a f/u > 6 months	Extrahepatic metastases, R2 resection, RFA, died within 90 days after surgery, repeated LR
Narita,2015, [30]Japan	2007–2009	PC	184	-	6	49.5	22.3	-	R0 LR for CRLM	Extrahepatic recurrence within 6 months, R1/R2 resection
Sakai,2021 [16],Japan	2001–2016	RC	229	-	12	73.4	42.4	-	Initial LR for CRLM	R2 resection
Sun,2014 [31],China	2000–2013	PC	152	22	6	63.1	24.9	-	-	-
Tabchouri,2018 [32],France	2000–2016	PC	273	41	6	72	22.8	-	Curative-intent LR for CRLM	A f/u < 6 months
Tanaka,2014 [33], Japan	1992–2011	RC	405	31	4	40.7	8.6	27.4	Curative resection for CRLM	R2 resection
Viganò,2014 [34], Italy, Multicenter	1998–2009	PC	6025	34.4	6	45.4	10.6	26.9	LR for CRLM, a f/u > 6 months	R2 resection, f/u < 6 months, two-stage LR, operative mortality
Viganò,2022 [12], Italy	2004–2017	PC	484	34	3	75.2	11.6	17.3	LR for CRLM	Repeated LR, died within 90 days, R2 resection, loss to f/p
Watanabe,2020 [35], Japan	2004–2016	PC	643	44.2	6	44.3	20.7	24	Initial LR for CRLM	R2 resection
Wong,2022 [36], Australia	2007–2017	PC	194	85.3	6	74.7	29.9	28.8	Initial curative‐intent LR for CRLM, a f/u > 6 months	R2 resection, a f/u < 6 months, died within 30 days
Yamashita,2011 [37], Japan	1986–2007	RC	121	-	12	67.8	43.0	20	Initial curative‐intent LR for CRLM	RFA or MCT

*ER* early recurrence, *OS* overall survival, *PC* prospective cohort, *RC* retrospective cohort, *LR* liver resection, *CRLM* colorectal liver metastases, *NAC* neoadjuvant chemotherapy, *NAR* neoadjuvant radiotherapy, f/u follow up *RFA* radiofrequency ablation, *CRLOM* colorectal liver oligometastases, *PD* progressive disease, *MCT* microwave coagulation therapy; any missing or not applicable parts were marked with ‘‘–’

Continuous variables were summarized using median and interquartile range values, while categorical variables were expressed as counts and percentages. In cases where RR was unavailable, we either convert OR to RR or employed the events and patients counts in both exposed and non-exposed groups to calculate RR. By using standardized forms, two authors (YT, YQW) independently extracted the data from each eligible study. Disagreements were resolved by consensus or discussion with the third person (NYW).

### Risk-of-bias assessment

To evaluate the risk-of-bias (RoB) at the study level, the Quality in Prognostic Factor Studies (QUIPS) tool was employed. This tool has six domains, with each domain assigned a RoB rating categorized as high, moderate, or low [38]. Studies were deemed to have low RoB if all domains were rated as low RoB or only one domain scored moderate RoB. Conversely, studies were classified as high RoB if at least one domain scored high RoB or if three or more domains scored moderate RoB. The remaining studies were attributed a moderate RoB rating.

### Statistical analysis

The primary outcomes of the study focused on the RRs depicting the association between ER and prognostic factors. When available, preference was given to the most adjusted effect estimate, specifically opting for the Cox multivariable coefficient over the univariable estimate. Subsequently, all pooled outcomes were derived utilizing a random-effects model (Mantel–Haenszel method). The magnitude of the summary effects was graphically represented through forest plots.

Between-study heterogeneity was assessed utilizing the I^2^ statistical estimate, with an I^2^ value > 50% regarded as severe heterogeneity [39]. Consequently, subgroup analyses were executed to identify potential sources of heterogeneity. Assessment of reporting bias was undertaken through funnel plots and the Egger's test, specifically for prognostic factors identified in over 10 studies. A *P* value below 0.1 was deemed indicative of significant publication bias, prompting the execution of Trim and Fill analysis in such instances. Additionally, sensitivity analysis was performed by switching to fixed-effects models to test the robustness of the conclusions.

All statistical analyses were conducted using Review Manager software (Version 5.4) and Stata software (version 14.1). A significant two-way *P* value for comparison was defined as *P* < 0.05.

### Evidence strength assessment

The grading of evidence strength for the identified associations in observational studies was based on the following criteria: Egger's test *P* value > 0.1, a cumulative population > 1000, and I^2^ < 50%. The association attained Class I (high-quality) evidence status when all three conditions were satisfied simultaneously. If two out of these three conditions were met, the association was categorized as Class II (moderate-quality) evidence. Furthermore, class III (moderate-quality) evidence was conferred upon an association when only one of the three conditions was fulfilled. Conversely, the absence of satisfaction for all of these three conditions designated an association as Class IV (low-quality) evidence [40].

## Result

### Study selection

**Fig. 1:**
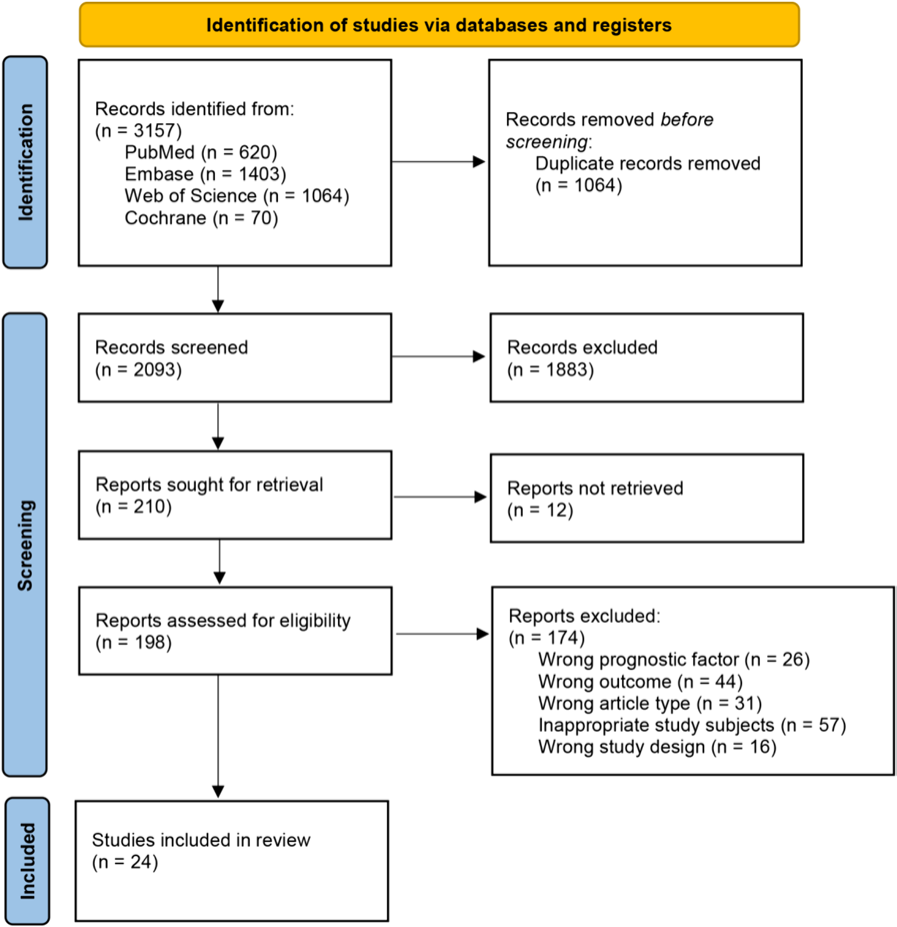
PRISMA flowchart version 2020

Our initial search strategy identified a total of 3157 pertinent studies, of which 1064 were removed due to duplication. Following the preliminary screening of titles and abstracts, 1883 abstracts were excluded because they did not meet the inclusion criteria. Furthermore, 12 reports were inaccessible, and 198 potentially relevant articles underwent a thorough review in full text. Ultimately, 174 articles were excluded for diverse reasons and 24 selected studies were included [12, 14–16, 18–37], as illustrated in the PRISMA flow diagram (Fig. 1).

### Study characteristics

This review included 24 studies, comprising 12,705 patients who underwent LR for CRLM, with a comprehensive summary presented in Table 1. Among these, twelve studies adopted a prospective cohort design [12, 18, 21, 22, 28–32, 34–36], with the remaining adopting a retrospective cohort approach [14–16, 19, 20, 23–27, 33, 37]. The publication years of the studies spanned from 2007 to 2023. In terms of geographical distribution, 15 studies originated from Asia [14–16, 19, 20, 23, 24, 26, 27, 29–31, 33, 35, 37], 7 from Europe [12, 18, 22, 25, 28, 32, 34], 1 from Australia [36], and the other one from the United States [21]. The recruitment period ranged from 1986 to 2020, with the median duration of follow-up ranging varying from 22 to 86.3 months.

### Definition of early recurrence

The definition of ER exhibited variation among the studies. Twelve studies defined ER as six-month following surgery [15, 23, 24, 26, 28–32, 34–36], while five studies utilized 12 months as the cutoff for ER [16, 21, 25, 27, 37]. The ER rate ranged from 8.6% to 54.3%, and the pooled ER rate was 30.2% (95% CI, 24.1%–36.4%), indicating substantial heterogeneity across the studies (I^2^ = 98%, *P* < 0.001) (Fig. S1). Additionally, Viganò et al. applied a 3-month threshold to define very early recurrence (VER), with 11.6% of patients experiencing VER [12]. The overall CRLM recurrence rate was reported to be between 40.7% and 78.8%. For those patients who underwent early recurrence (ER), the 5-year overall survival (OS) spanned from 11.1% to 45.0% (Table 1).

### Prognostic factors

A total of 22 potential prognostic factors were identified before the study, categorized into patient-related factors, primary tumor factors, liver metastasis factors, and treatment-related factors. The characteristics of these prognostic factors in the ER group were presented in Tables 2, 3, 4 and 5. As shown, the median age among patients with ER ranged from 55 to 66 years, and the proportion of males varied between 44.6% and 67.3%. Regarding primary tumor characteristics, poor tumor differentiation in the ER group ranged from 5.6% to 66.7%, and 42.3% to 55.7% of patients had LNM. As factors of liver metastases, bilobar distribution was noted in 26.7% to 74.2% of the ER group, and Jung et al. reported that up to 93.3% had synchronous metastases [24]. As reported, 11.5% to 63.3% of patients with ER had positive surgical margins, and approximately 30% to 80% of patients received chemotherapy.

**Table 2:** Prognostic factors of patient characteristics in ER group

First author, year	Median age (years)	Male (%)	CEA	CA199
Bhogal,2015 [18]	-	-	-	-
Chen,2022 [19]	55.0	67.3	-	-
Dai,2021 [15]	62.7	66.7	24.2%, > 100 ng/mL	30.3%, > 320 U/ml
Deng,2023 [20]	-	66.8	5.0%, > 200 ng/mL	-
Finkelstein,2008 [21]	-	-	-	-
Imai,2016 [22]	-	61.5	44.8%, > 10 ng/mL	29.8%, > 60 U/ml
Inoue,2020 [23]	66.0	64.8	14.9 ng/mL, med	27.7 U/ml, med
Jung,2016 [24]	-	66.7	53.3%, ≥ 50 ng/mL	-
Kaibori,2012 [14]	-	59.2	50%, > 6 ng/mL	35.2%, > 30 ng/dl
Lalmahomed,2015 [25]	63.0	63.4	-	-
Lin,2018 [26]	-	65.3	52.1%, > 10 ng/mL	37.5%, > 35 U/ml
Liu,2015 [27]	-	44.6	47.8%, > 200 ng/mL	-
Malik,2007 [28]	62.0	55.8	25.0 ng/mL, med	34.0 U/ml, med
Mao,2017 [29]	57.0	56.3	39.1%, > 30 ng/mL	-
Narita,2015 [30]	56.5	53.3	79.8 ng/mL, med	-
Sakai,2021 [16]	-	-	-	-
Sun,2014 [31]	58.2	50.0	79.1 ng/mL, med	-
Tabchouri,2018 [32]	-	-	-	-
Tanaka,2014 [33]	61.6	51.4	235.3 ng/mL, med	-
Viganò,2014 [34]	-	58.2	10.2%, > 200 ng/mL	-
Viganò,2022 [12]	-	55.4	8.9%, > 200 ng/mL	-
Watanabe,2020 [35]	62.0	55.0	11.1 ng/mL, med	18.4 U/ml, med
Wong,2022 [36]	66.6	50.0	-	-
Yamashita,2011 [37]	59.0	59.6	26.9%, > 50 ng/mL	-

**Table 3:** Prognostic factors of primary tumor characteristics in ER group

First author, year	Poor tumor diff-erentiation (%)	LNM (%)	T3-4 (%)	Rectal tumor (%)
Bhogal,2015 [18]	-	-	-	30.1
Chen,2022 [19]	28.8	77.9	95.2	47.1
Dai,2021 [15]	-	77.3	-	25.8
Deng,2023 [20]	34.9	83.0	94.2	44.8
Finkelstein,2008 [21]	30.0	70.0	-	33.3
Imai,2016 [22]	-	62.3	80.2	24.6
Inoue,2020 [23]	-	81.8	-	42.0
Jung,2016 [24]	66.7	63.3	63.3	40.0
Kaibori,2012 [14]	5.6	68.5	87.0	29.6
Lalmahomed,2015 [25]	-	59.8	84.1	28.0
Lin,2018 [26]	28.6	72.7	-	34.7
Liu,2015 [27]	-	54.6	47.5	47.6
Malik,2007 [28]	-	58.1	-	-
Mao,2017 [29]	28.7	81.6	96.6	-
Narita,2015 [30]	-	63.3	-	36.7
Sakai,2021 [16]	-	-	-	-
Sun,2014 [31]	-	80.0	-	56.7
Tabchouri,2018 [32]	-	77.8	-	-
Tanaka,2014 [33]	11.4	-	-	34.3
Viganò,2014 [34]	-	68.8	90.8	35.6
Viganò,2022 [12]	-	67.9	83.9	28.6
Watanabe,2020 [35]	6.1	76.3	87.8	37.4
Wong,2022 [36]	-	74.1	-	31.0
Yamashita,2011 [37]	-	42.3	65.4	32.7

**Table 4:** Prognostic factors of liver metastases characteristics in ER group

First author, year	Synchronous metastases (%)	More metastases (%)	Diameter (median, cm)	Bilobar- distribution (%)	Extrahepatic metastases (%)	Initial un- resectable (%)
Bhogal,2015 [18]	-	-	-	-	-	-
Chen,2022 [19]	91.3	-	3.0	60.6	11.5	-
Dai,2021 [15]	78.8	42.4	2.7	40.9	-	-
Deng,2023 [20]	-	66.8	-	47.7	14.1	-
Finkelstein,2008 [21]	66.7	40.0	-	26.7	-	-
Imai,2016 [22]	71.8	54.0		66.7	23.0	45.2
Inoue,2020 [23]	63.6	64.8	3.2	-	-	-
Jung,2016 [24]	93.3	56.7	-	46.7	-	-
Kaibori,2012 [14]	68.5	44.4	-	44.4	-	-
Lalmahomed,2015 [25]	-	-	2.8	32.9	-	-
Lin,2018 [26]	63.3	14.3	-	34.7	-	-
Liu,2015 [27]	22.2	60.6	-	57.3	45.5	-
Malik,2007 [28]	47.7	36.0	4.5	-	-	-
Mao,2017 [29]	80.5	82.0	3.0	50.6	-	69.0
Narita,2015 [30]	-	66.7	-	43.3	-	-
Sakai,2021 [16]	-	-	-	-	-	-
Sun,2014 [31]	68.3	-	4.1	-	-	-
Tabchouri,2018 [32]	-	-	-	-	-	-
Tanaka,2014 [33]	74.3	54.3	5.2	74.2	14.3	-
Viganò,2014 [34]	63.4	29.1	-	39.8	8.1	20.7
Viganò,2022 [12]	78.6	92.9	-	-	23.2	-
Watanabe,2020 [35]	75.6	-	3.0	-	-	-
Wong,2022 [36]	70.6	-	3.1	44.8	-	-
Yamashita,2011 [37]	82.7	36.5	3.7	28.8	-	-

**Table 5:** Prognostic factors of therapy characteristics in ER group

First author, year	Laparoscopic resection (%)	Simultaneous resection (%)	Major hepatectomy (%)	R1 resection (%)	Preoperative chemotherapy (%)	Postoperative chemotherapy (%)	Blood transfusion (%)	Postoperative complications (%)
Bhogal,2015 [18]	-	-	-	-	-	92.3	-	-
Chen,2022 [19]	-	73.1	84.6	46.2	61.5	59.6	-	59.6
Dai,2021 [15]	-	-	-	-	-	-	-	-
Deng,2023 [20]	15.8	-	57.7	32.8	56.0	63.1	23.2	53.9
Finkelstein,2008 [21]	-	-	13.3	3.3	-	-	-	-
Imai,2016 [22]	-	-	57.1	53.2	100	83.3	38.9	22.2
Inoue,2020 [23]	-	17.0	-	21.8	34.1	38.8	18.4	28.4
Jung,2016 [24]	0.0	30.0	20.0	63.3	-	40.0	-	-
Kaibori,2012 [14]	-	-	37.0	24.1	37.0	55.6	37.0	37.0
Lalmahomed,2015 [25]	-	32.9	-	-	-	-	-	-
Lin,2018 [26]	-	-	-	-	55.1	71.4	-	-
Liu,2015 [27]	-	51.9	50.0	45.0	58.3	48.7	61.3	-
Malik,2007 [28]	-	-	-	34.9	-	-	-	-
Mao,2017 [29]	4.6	60.9	-	42.5	74.7	-	18.4	14.9
Narita,2015 [30]	-	56.7	33.3	-	80.0	-	30.0	-
Sakai,2021 [16]	-	-	-	32.0	-	-	-	-
Sun,2014 [31]	-	11.7	28.3	-	50.0	60.0	-	33.3
Tabchouri,2018 [32]	-	-	-	-	-	-	-	-
Tanaka,2014 [33]	-	-	-	-	34.3	68.6	-	-
Viganò,2014 [34]	1.7	-	-	-	-	47.9	24.4	-
Viganò,2022 [12]	-	10.7	10.7	73.2	-	-	-	33.9
Watanabe,2020 [35]	-	-	17.6	11.5	38.9	39.7	13.7	-
Wong,2022 [36]	-	-	39.7	34.5	86.2	74.1	-	-
Yamashita,2011 [37]	-	-	34.6	-	-	-	32.7	-

### Assessment on risk-of-bias

The results of the RoB assessment were presented in Table 6. Employing the QUIPS tool and the criteria described above, 17 studies received a classification of low overall RoB, whereas 5 studies were assigned a moderate RoB rating. Notably, two studies were excluded due to high RoB at this stage [33, 34].

**Table 6:** Risk of bias assessment using QUIPS tool Risk of bias assessment using QUIPS tool

Study	1.Study participation	2.Study attrition	3. PF measurement	4. Outcome measurement	5. Adjustment for other PF	6. Statistical analysis and reporting	Overall
Bhogal,2015, [18]	Mod^a^	Mod^c^	Low	Low	Low	Low	Mod
Chen,2022, [19]	Low	Low	Low	Low	Low	Low	Low
Dai,2021, [15]	Low	Low	Low	Low	Low	Low	Low
Deng,2023, [20]	Low	Mod^c^	Low	Low	Low	Low	Low
Finkelstein,2008, [21]	Mod^a^	Mod^c^	Low	Low	Low	Low	Mod
Imai,2016, [22]	Low	Mod^c^	Low	Low	Low	Low	Low
Inoue,2020 [23]	Low	Mod	Low	Low	Low	Low	Low
Jung,2016, [24]	Low	Mod^c^	Low	Low	Low	Low	Low
Kaibori,2012 [14]	Mod^b^	Mod^c^	Low	Low	Low	Low	Mod
Lalmahomed,2015, [25]	Low	Low	Low	Low	Mod^f^	Low	Low
Lin,2018, [26]	Low	Low	Low	Low	Low	Low	Low
Liu,2015, [27]	Mod^a^	Mod^c^	Low	Low	Low	Low	Mod
Malik,2007, [28]	Low	Mod^c^	Low	Low	Low	Low	Low
Mao,2017, [29]	Low	Mod^c^	Low	Low	Low	Low	Low
Narita,2015, [30]	Low	Mod^c^	Low	Low	Mod^f^	Low	Mod
Sakai,2021, [16]	Low	Mod^c^	Low	Low	Low	Low	Low
Sun,2014, [31]	Mod^a^	Low	Low	Low	Low	Low	Low
Tabchouri,2018, [32]	Low	Low	Low	Low	Low	Low	Low
Tanaka,2014, [33]	Mod^a^	Mod^c^	Low	Mod^e^	Low	Low	High
Viganò,2014, [34]	Mod^b^	Mod^c^	Mod^d^	Low	Low	Low	High
Viganò,2022, [12]	Low	Low	Low	Low	Low	Low	Low
Watanabe,2020, [35]	Low	Low	Low	Low	Low	Low	Low
Wong,2022, [36]	Low	Low	Low	Low	Low	Low	Low
Yamashita,2011, [37]	Low	Low	Low	Low	Low	Low	Low

*PF* prognostic factor, *Mod* moderate

^a^Lacks inclusion and exclusion criteria

^b^Lacks the baseline of study sample

^c^Lacks reporting of exact study

### Meta-analysis for prognostic factors

A total of 21 studies, involving 5791 patients, met the eligibility criteria for the meta-analysis. One study was omitted from consideration due to its utilization of VER (3 months) as the outcome, and two additional studies were excluded on account of high RoB.

**Fig. 2:**
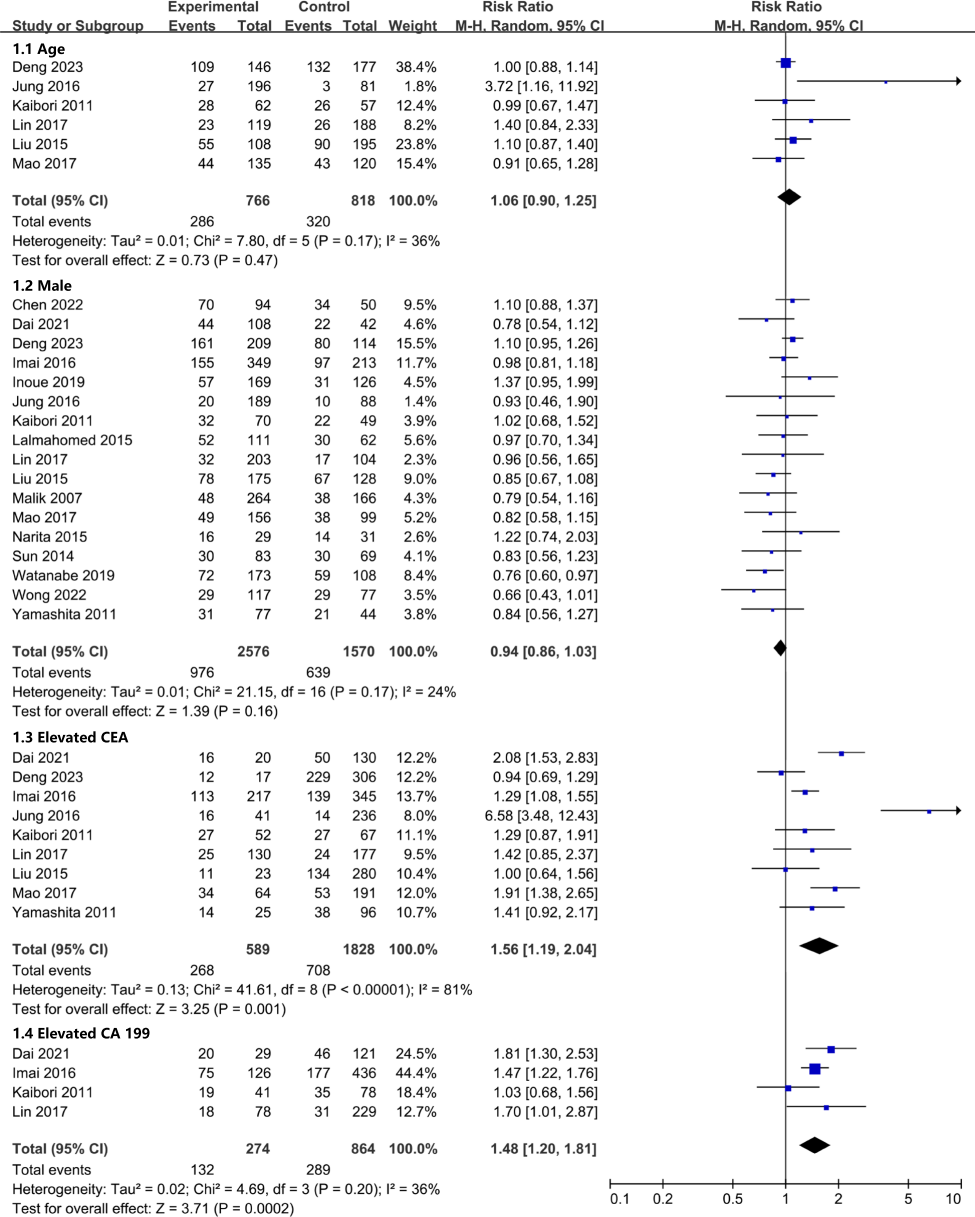
Meta-analyses of association between patient characteristics and ER

**Fig. 3:**
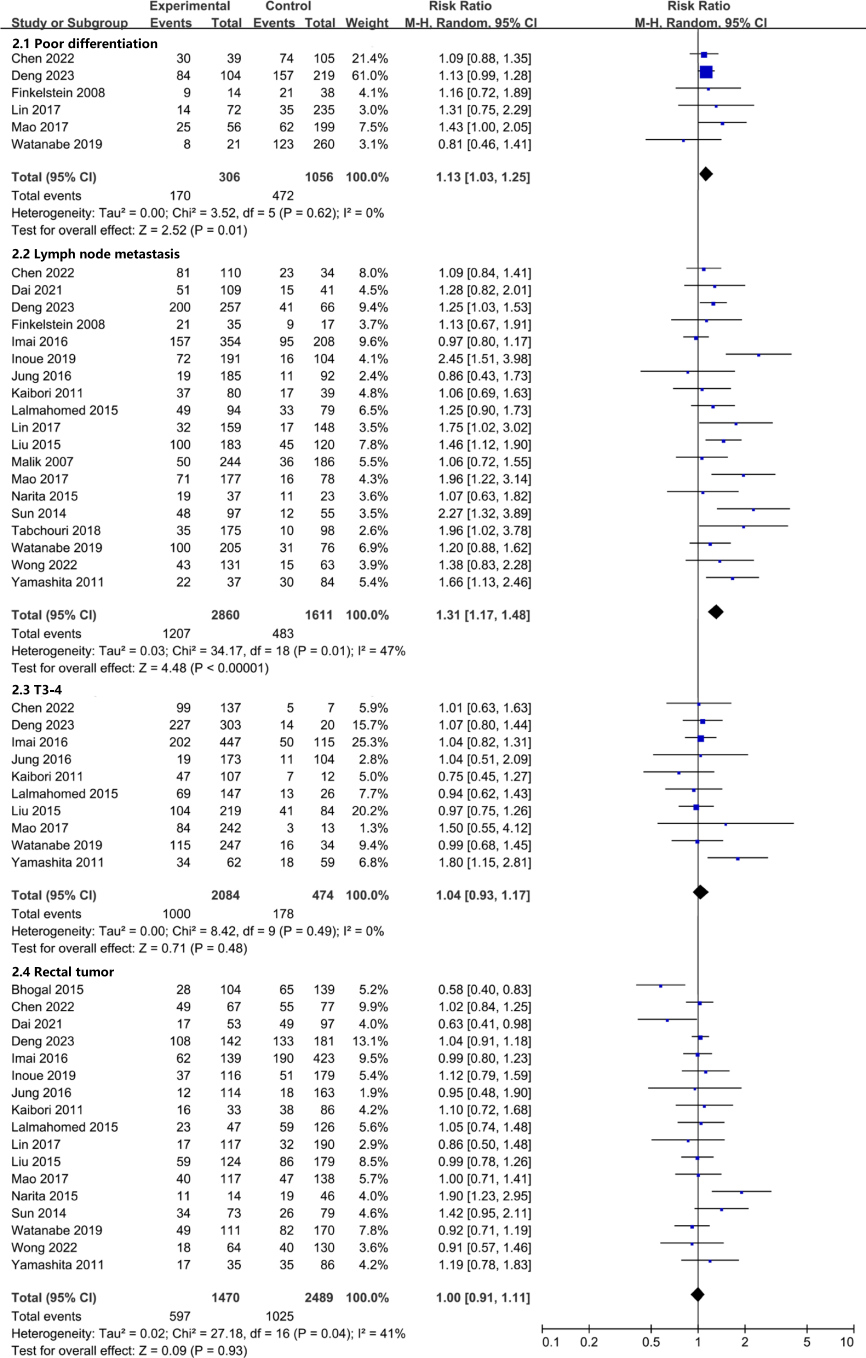
Meta-analyses of association between primary tumor factors and ER

**Fig. 4:**
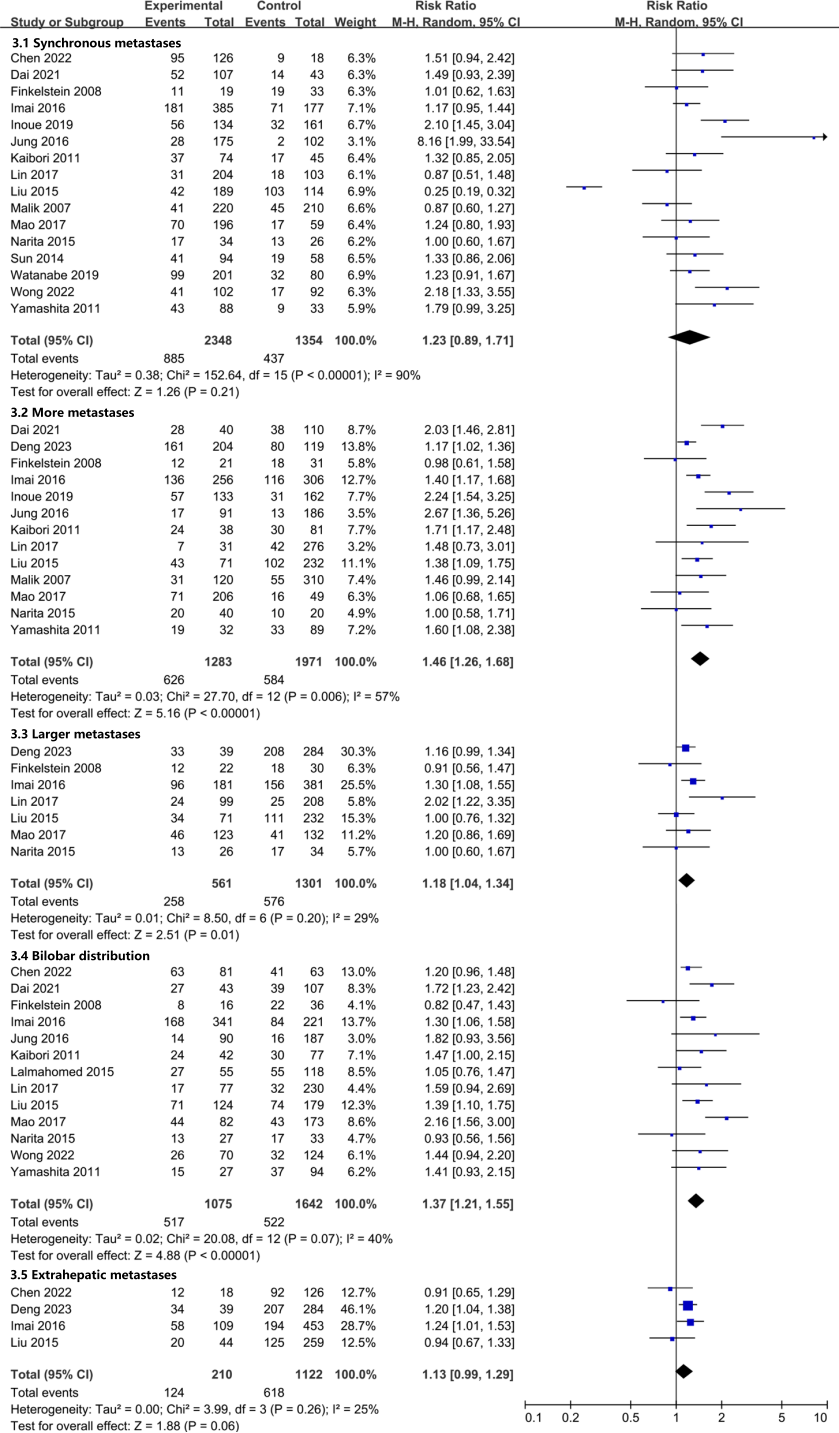
Meta-analyses of association between liver metastases factors and ER

**Fig. 5:**
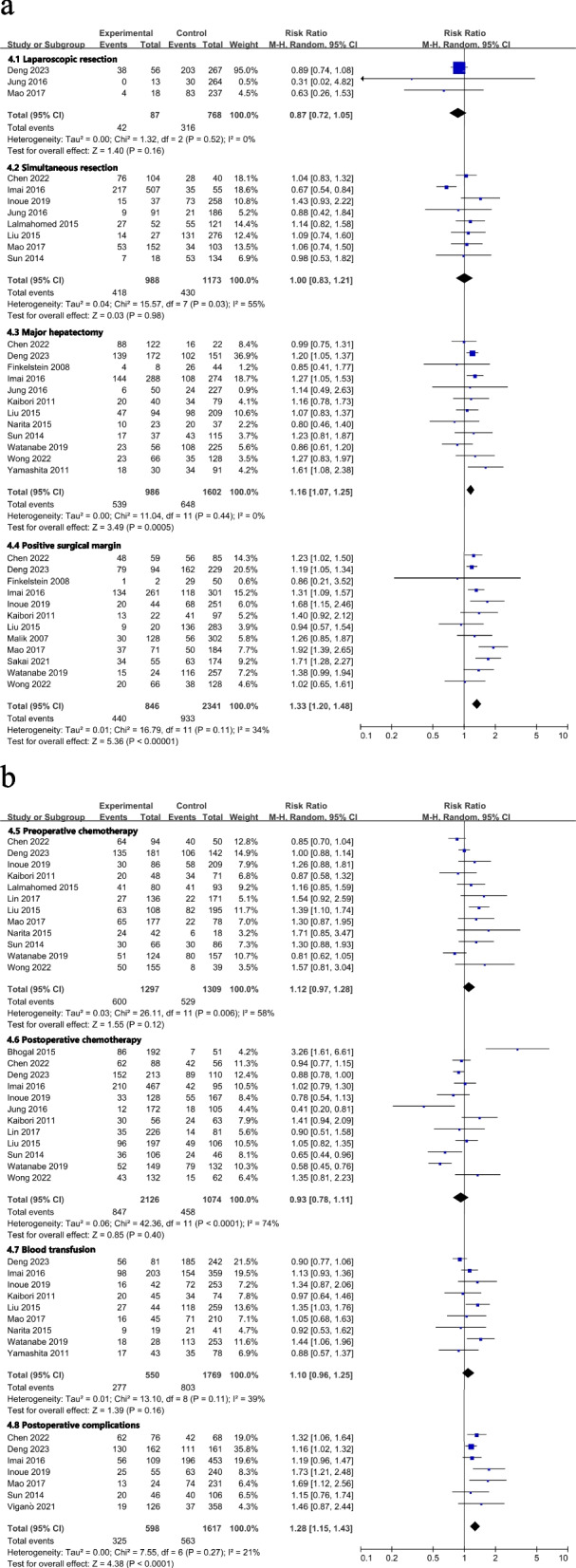
**a** Meta-analyses of association between therapeutic factors and ER. **b** Meta-analyses of association between therapeutic factors and ER

All results graphically depicted using forest plots, illustrated in Figs. 2, 3, 4 and 5. Patient- related factors such as age and male gender exhibited no correlation with ER. Elevated concentrations of preoperative CEA (RR, 1.56; 95% CI, 1.19–2.04; I^2^ = 81%) and CA199 (RR, 1.48; 95% CI, 1.20–1.81; I^2^ = 36%) were identified as potential risk factors for ER (Fig. 2). Besides, primary tumor factors associated with an increased hazard of ER encompassed poor differentiation (RR, 1.13; 95% CI, 1.03–1.25; *I*^*2*^ = 0%) and LNM (RR, 1.31; 95% CI, 1.17–1.48; *I*^*2*^ = 47%) (Fig. 3). Concerning liver metastases, an elevated risk of ER was associated with factors such as a higher number of metastases (RR, 1.46; 95% CI, 1.26–1.68; *I*^*2*^ = 57%), larger metastases (RR, 1.18; 95% CI, 1.04–1.34; *I*^*2*^ = 29%), and bilobar distribution (RR, 1.37; 95% CI, 1.21–1.55; *I*^*2*^ = 40%) (Fig. 4). Regarding therapeutic factors, major hepatectomy (RR, 1.16; 95% CI, 1.07–1.25; *I*^*2*^ = 0%), positive surgical margins (RR, 1.33; 95% CI, 1.20–1.48; *I*^*2*^ = 34%), and postoperative complications (RR, 1.28; 95% CI, 1.13–1.44; *I*^*2*^ = 30%) have been recognized as risk factors associated with ER (Fig. 5a and b).

However, the stage and location of the primary tumor, synchronous metastases, extrahepatic metastases, laparoscopic surgery, preoperative or postoperative chemotherapy, and blood transfusion were not found to be statistically associated with ER. All the above results are presented in Table 7.

**Table 7:** Summary of meta-analysis results and evidence quality

Outcome	Studies	Participants	RR & 95%CI	*P* value	I^2^	Egger's test *P* value	Class of Evidence
1. Patient characteristic							
1.1 Age	6	1584	1.06 [0.90, 1.25]	0.47	36%	-	Class II
1.2 Male	17	4146	0.94 [0.86, 1.03]	0.16	24%	0.185	Class I
1.3 Elevated CEA	9	2417	1.56 [1.19, 2.04]	0.001	81%	-	Class II
1.4 Elevated CA199	4	1138	1.48 [1.20, 1.81]	< 0.001	36%	-	Class II
2. Primary tumor characteristics							
2.1 Poor differentiation	6	1362	1.13 [1.03, 1.25]	0.01	0%	-	Class I
2.2 Lymph node metastasis	19	4471	1.31 [1.17, 1.48]	< 0.001	47%	0.035	Class II
2.3 T3-4	10	2558	1.04 [0.93, 1.17]	0.48	0%	0.623	Class I
2.4 Rectal tumor	17	3959	1.00 [0.91, 1.11]	0.93	41%	0.897	Class I
3. Liver metastases characteristics							
3.1 Synchronous metastases	16	3702	1.23 [0.89, 1.71]	0.21	90%	0.121	Class III
3.2 More metastases	13	3254	1.46 [1.26, 1.68]	< 0.001	57%	0.206	Class II
3.3 Larger metastases	7	1862	1.18 [1.04, 1.34]	0.01	29%	-	Class I
3.4 Bilobar distribution	13	2717	1.37 [1.21, 1.55]	< 0.001	40%	0.811	Class I
3.5 Extrahepatic metastases	4	1332	1.13 [0.99, 1.29]	0.06	25%	-	Class I
4. Surgical procedures and operative outcome							
4.1 Laparoscopic resection	3	855	0.87 [0.72, 1.05]	0.16	0%	-	Class II
4.2 Simultaneous resection	8	2161	1.00 [0.83, 1.21]	0.98	55%	-	Class II
4.3 Major hepatectomy	12	2588	1.16 [1.07, 1.25]	< 0.001	0%	0.329	Class I
4.4 Positive surgical margin	12	3187	1.33 [1.20, 1.48]	< 0.001	34%	0.505	Class I
4.5 Preoperative chemotherapy	12	2606	1.12 [0.97, 1.28]	0.12	58%	0.074	Class III
4.6 Postoperative chemotherapy	12	3200	0.93 [0.78, 1.11]	0.40	74%	0.616	Class II
4.7 Blood transfusion	9	2319	1.10 [0.96, 1.25]	0.16	39%	-	Class I
4.8 Postoperative complications	6	1731	1.28 [1.13, 1.44]	< 0.001	30%	-	Class I

The CRS ranges from 0 to 5 points, with 1 point assigned for each of the following: LNM of the primary tumor, the interval < 1 year from primary tumor resection to the detection of liver metastasis, preoperative CEA > 200 ng/ml, more than one liver tumor, and largest tumor > 5 cm [41]. The combination of RRs in three studies showed that CRS > 2 had the potential to increase the risk of ER (RR, 1.44; 95% CI, 1.17–1.77; I^2^ = 0%; Egger’s *P* value = 0.232) (Fig. S2).

### Reporting bias

**Fig. 6:**
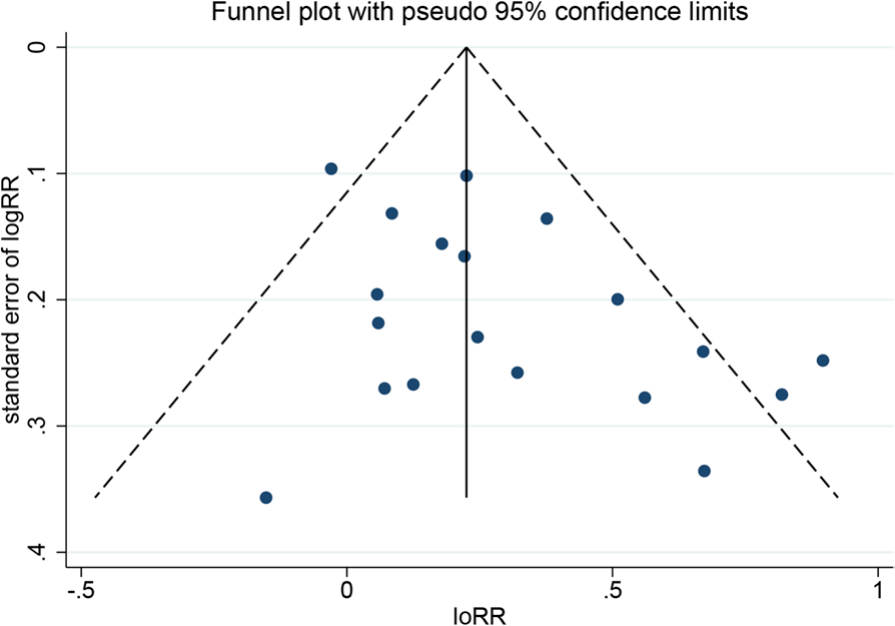
Funnel plot of ER in groups with and without LNM in the primary tumor

**Fig. 7:**
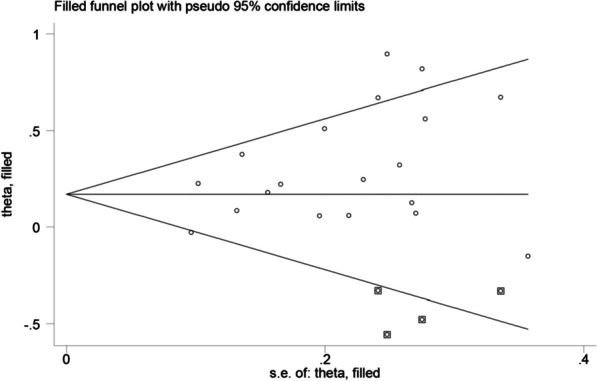
Trim and Fill analysis of the effect of LNM on ER. The squares represent the adjusted studies

Reporting bias was evaluated by funnel plot and Egger's test. Our results comparing the ER rates between groups with and without LNM of primary tumor revealed an asymmetric funnel, with a *P* value of 0.035 for the Egger's test (Fig. 6). By filling 4 studies using the Trim and Fill method, the recalculated pooled RR was 1.20, 95% CI (1.06, 1.37) (Fig. 7), which was not significantly changed from the initial estimate (RR, 1.31; 95% CI, 1.17–1.48). Therefore, the presence of publication bias has little significant effect on the overall finding.

### Study quality

Using the rating rules mentioned above, no evidence was rated as Class IV. High-quality (Class I) evidence showed that poor differentiation of CRC, larger and bilobar-distributed liver metastases, major hepatectomy, positive surgical margins, and postoperative complications were factors linked to an elevated hazard of ER. Among other meaningful prognostic factors, elevated levels of CEA and CA199, LNM, and a higher number of liver metastases were rated as Class II (Table 7).

### Subgroup analyses

**Fig. 8:**
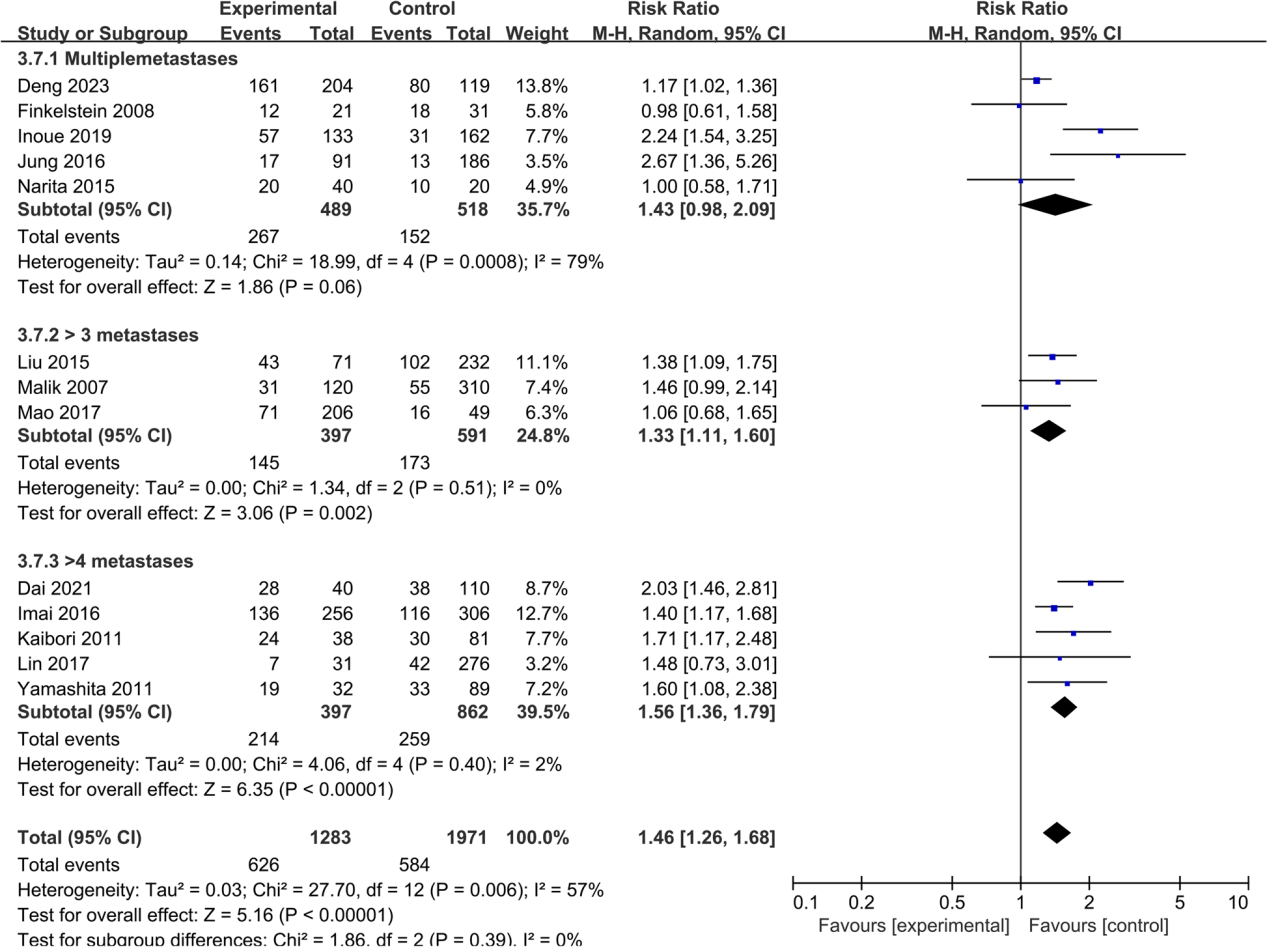
Subgroup analyses of more metastases

Among the prognostic factors analyzed, an elevated number of metastases was reported of having high heterogeneity (I^2^ > 50%). Subgroup analyses were conducted, employing diverse thresholds for defining an increased number of metastases, categorized as multiple, > 3, and > 4 metastases. As shown in Fig. 8, all subgroup analyses showed significant differences in the ER rate between cases with more and fewer metastases. Notably, the subgroup of multiple metastases showed great heterogeneity (I^2^ = 79%), whereas the other two groups did not. Therefore, we found that the divergent definitions of “multiple” across different articles constituted the primary source of heterogeneity.

### Sensitivity analyses

We performed sensitivity analysis by switching to fixed-effects models on all variables. The results were consistent across all variables except in the case of preoperative chemotherapy, wherein a fixed-effects model revealed an association with diminished risk of ER (RR, 1.11; 95% CI, 1.02–1.21; I^2^ = 58%) (Fig. S3). However, this result was deemed unreliable and excluded.

## Discussion

This is the first-ever published meta-analysis summarizing prognostic factors associated with ER following LR for CRLM. Specifically, most of the studies used a postoperative interval of 6 months to define ER, which is earlier than the ER definition for other tumors in the liver. For example, hepatocellular carcinoma (HCC) and intrahepatic cholangiocarcinoma (iCCA) often use 1 year or 2 years as a cutoff value [42–44]. This review reveals that following LR for CRLM, the occurrence of ER is approximated at 30.2% (95% CI, 24.1%–36.4%). However, evidence shows that the 5-year OS rate after ER of CRLM ranges from 11.1% to 45%, while that of iCCA is only 8–11.6% [43–45]. This suggests that postoperative ER of CRLM is more common but the prognosis is relatively better, compared with other intrahepatic tumors. Furthermore, with the development of surgical techniques and minimally invasive local treatment strategies, CRLM patient is more likely to undergo re-resection and/or ablation after recurrence, and the 5-year OS after repeat hepatectomy is as high as 50% [46].

After pooling data from 21 studies involving 5791 patients, this meta-analysis identified ten prognostic factors that could play a crucial role in ER across four domains: patient-related factors, primary tumor characteristics, liver metastases attributes, and therapeutic factors.

As patient-related factors, elevated levels of preoperative CEA and CA199 were identified as potential risk factors for ER. However, the evidence was classified as level II due to high heterogeneity, which was attributed to varying cutoff values in different studies. Studies indicated that postoperative serum molecular markers had stronger predictive potential. Even within the normal limits, higher levels of postoperative CA199 were effective in predicting ER [15, 47, 48].

For primary tumor factors, the analysis of aggregated RR values indicated a heightened risk of ER associated with poor differentiation and LNM, both indicative of a more advanced tumor stage. The impact of LNM on ER was categorized as class II evidence due to reporting bias, but the Trim and Fill analysis indicated that the presence of publication bias had no substantial influence on the overall findings. Besides, previous investigations have validated that individuals with LNM in primary tumors exhibit an adverse OS and progression-free survival (PFS) [49–51]. This reveals the significance of pathological characteristics of primary colorectal tumors in the prognosis assessment following LR. Particularly for metachronous liver metastases, defined as liver metastases discovered after primary tumor surgery, these primary tumor factors may assist surgeons in identifying patients who would derive greater benefits from LR [52, 53].

Characteristics of liver metastases, such as increased size, number, and bilobar distribution, have been recognized as potential risk factors associated with ER. Tumor size and number were frequently treated as dichotomous variables, with varying cutoff values, leading to heterogeneity across studies. However, these two variables can be used to calculate the tumor burden score (TBS) [TBS^2^ = (maximum tumor diameter)^2^ + (number of liver lesions)^2^], and the predictive efficacy of this index has been proved to exhibit higher specificity and sensitivity compared to relying solely on tumor size and number in patients with CRLM [54, 55]. Preoperative radiological imaging can provide first indications about the risk of ER, especially gadoxetate disodium-enhanced magnetic resonance imaging (EOB-MRI) can provide greater sensitivity [56]. Furthermore, the implementation of intraoperative hepatic ultrasonography (IOUS) has been reported to identify more occult hepatic lesions missed by preoperative imaging, thereby potentially mitigating the risk of ER [57, 58].

In terms of therapeutic factors, major hepatectomy, positive surgical margins, and postoperative complications have been identified to increase the risk of ER. But no statistically significant difference was observed in the impact on ER between laparoscopic and open hepatectomy, suggesting the viability of the laparoscopic approach. Major hepatectomy is traditionally defined as the resection of three or more liver segments [59]. Indeed, the presence of more and larger, and bilobar-distributed metastases mentioned above not only represents worse tumor behavior but also increases the probability of occult intrahepatic spread and affects the radicality of the treatment. Consequently, to ensure the complete removal of all lesions, more extensive liver resection may be performed, leaving little room for salvageability [60]. Besides, R1 resection has been identified as a risk factor, implying that overlooked lesions and residual microscopic tumor cells left after surgery contribute to ER. However, it remains uncertain whether recurrence at the surgical margin or the emergence of new metastases are the primary contributors to ER in patients with positive surgical margins. Severe postoperative complications could potentially extend the immunosuppression induced by major surgeries and delay the initiation of adjuvant chemotherapy [14].

The current study failed to demonstrate the benefit of preoperative or postoperative chemotherapy in preventing ER. The reason is that, on the one hand, patients with more advanced tumors and R1 resection have a greater tendency to receive adjuvant therapy, on the other hand, the effects of different regimens and cycles of adjuvant treatment are combined. But a study revealed that > 1 chemotherapy line and progression of disease during last-line chemotherapy, were identified as independent predictors of ER, suggesting that the response to chemotherapy was more important than the chemotherapy itself [22]. Therefore, the cooperation between surgeons and oncologists is essential, especially when aggressive indications are present.

In addition to individual prognostic factors, the CRS combines tumor markers, primary tumor factors, and liver metastasis factors and was widely adopted for the prognosis of CRLM patients. In this study, class I evidence demonstrated that a CRS > 2 increase the risk of ER. Furthermore, several studies have utilized multiple prognostic factors to develop nomograms to better predict ER of CRLM [15, 19, 20]. However, the generalizability of these nomograms requires further research, and there is an urgent need to develop a universal ER risk prediction score.

When liver recurrence occurs, salvage resection had the potential to extend long-term survival [22]. However, it was noteworthy that the secondary resection rate was notably diminished in individuals with ER compared to those experiencing late recurrence, due to worsened condition status and potential surgical complications [26]. Additionally, studies indicated that the survival benefit associated with salvage resection disappeared within the subgroup of patients exhibiting more than two risk factors for ER [29]. For these patients, salvage treatment may accelerate disease progression and postoperative complications, thereby mitigating survival benefits rather than effectively controlling local recurrence. Therefore, the indications for salvage resection in patients with ER should be strictly controlled.

There are several limitations in this review. Initially, all the included studies are non-randomized control trials, introducing the possibility of confounding bias. Moreover, the combination of various ER definitions, ranging from 6 to 24 months, and amalgamation of diverse cutoff values for prognostic factors contributed to the substantial heterogeneity. In addition, only RRs were extracted and combined, not the hazard ratios (HRs), which were less persuasive due to the absence of time-related data. Despite these limitations, pooling evidence from available observational studies enabled us to synthesize relevant and generalizable prognostic factors.

## Conclusion

This review offers a consolidated summary of the prognostic factors associated with ER subsequent to LR for CRLM. These findings have the potential to enhance the efficacy of surveillance strategies, refine prognostic assessments, and guide judicious treatment decisions for CRLM patients with high risk of ER. Additionally, it is essential to undertake well-designed prospective investigations to examine additional prognostic factors and develop salvage therapeutic approaches for ER of CRLM.

### Supplementary Information

**Supplementary Material 1. **

**Supplementary Material 2. **

**Supplementary Material 3. **

**Supplementary Material 4. **

**Supplementary Material 5. **

## Data Availability

Yes, I have research data to declare. Data is provided within the manuscript or supplementary information files.

## Abbreviations

CA199: carbohydrate antigen 19–9
CEA: carcinoembryonic antigen
CI: confidence interval
CRC: colorectal cancer
CRLM: colorectal liver metastases
CRS: clinical risk score
ER: early recurrence
HCC: hepatocellular carcinoma
HR: hazard ratio
iCCA: intrahepatic cholangiocarcinoma
IOUS: intraoperative hepatic ultrasonography
LNM: lymph node metastases
LR: liver resection
OR: odds ratio
OS: overall survival
PFS: progression-free survival
PRISMA: Preferred Reporting Items for Systematic Reviews and Meta-analyses
QUIPS: Quality in Prognostic Factor Studies
RoB: risk-of-bias
RR: relative ratio
VER: very early recurrence
