# Supplementary material for: Prognostic factors associated with early recurrence following liver resection for colorectal liver metastases: a systematic review and meta-analysis
Source: BMC Cancer. 2024 Apr 8;24:426. doi: 10.1186/s12885-024-12162-4 (PMC11000331; doi:10.1186/s12885-024-12162-4)
Supplement: Supplementary file 2 — Supplementary Material 2. [file 12885_2024_12162_MOESM2_ESM.docx]

**Search strategy**

**Pubmed**

| No. | Query | Results |
| --- | --- | --- |
| 1 | "Colorectal Neoplasms"[Mesh] | 237,272 |
| 2 | (colorectal*[tiab] OR colon*[tiab] OR rectal*[tiab] OR rectum[tiab] OR sigmoid) AND (neoplas*[tiab] OR cancer*[tiab] OR carcinoma*[tiab] OR tumour[tiab] OR tumours[tiab] OR metasta*[tiab] OR malig*[tiab]) | 353,877 |
| 3 | ("Colorectal Neoplasms"[Mesh]) OR ((colorectal*[tiab] OR colon*[tiab] OR rectal*[tiab] OR rectum[tiab] OR sigmoid) AND (neoplas*[tiab] OR cancer*[tiab] OR carcinoma*[tiab] OR tumour[tiab] OR tumours[tiab] OR metasta*[tiab] OR malig*[tiab])) | 403,780 |
| 4 | "Liver Neoplasms"[Mesh] | 193,152 |
| 5 | (liver[tiab] OR hepatic*[tiab]) AND (neoplas*[tiab] OR cancer*[tiab] OR carcinoma*[tiab] OR tumour[tiab] OR tumours[tiab] OR metasta*[tiab] OR malig*[tiab]) | 230,583 |
| 6 | ("Liver Neoplasms"[Mesh]) OR ((liver[tiab] OR hepatic*[tiab]) AND (neoplas*[tiab] OR cancer*[tiab] OR carcinoma*[tiab] OR tumour[tiab] OR tumours[tiab] OR metasta*[tiab] OR malig*[tiab])) | 322,761 |
| 7 | "Surgical Procedures, Operative"[Mesh] | 3,538,914 |
| 8 | resection*[tiab] OR excision*[tiab] OR surgery*[tiab] | 1,798,911 |
| 9 | ("Surgical Procedures, Operative"[Mesh]) OR (resection*[tiab] OR excision*[tiab] OR surgery*[tiab]) | 4,394,291 |
| 10 | "Recurrence"[Mesh] | 200,682 |
| 11 | recurrence*[tiab] OR recrudescence*[tiab] OR relapse*[tiab] | 567,547 |
| 12 | (("Recurrence"[Mesh]) OR (recurrence*[tiab] OR recrudescence*[tiab] OR relapse*[tiab])) AND (earl*[tiab]) | 90,372 |
| 13 | (((("Colorectal Neoplasms"[Mesh]) OR ((colorectal*[tiab] OR colon*[tiab] OR rectal*[tiab] OR rectum[tiab] OR sigmoid) AND (neoplas*[tiab] OR cancer*[tiab] OR carcinoma*[tiab] OR tumour[tiab] OR tumours[tiab] OR metasta*[tiab] OR malig*[tiab]))) AND (("Liver Neoplasms"[Mesh]) OR ((liver[tiab] OR hepatic*[tiab]) AND (neoplas*[tiab] OR cancer*[tiab] OR carcinoma*[tiab] OR tumour[tiab] OR tumours[tiab] OR metasta*[tiab] OR malig*[tiab])))) AND (("Surgical Procedures, Operative"[Mesh]) OR (resection*[tiab] OR excision*[tiab] OR surgery*[tiab]))) AND ((("Recurrence"[Mesh]) OR (recurrence*[tiab] OR recrudescence*[tiab] OR relapse*[tiab])) AND (earl*[tiab])) | 620 |

**Embase**

| No. | Query | Results |
| --- | --- | --- |
| #1 | 'colorectal liver metastasis'/exp | 6668 |
| #2 | 'colorectal tumor'/exp | 469170 |
| #3 | ('colorectal*':ab,ti OR 'colon*':ab,ti OR 'rectal*':ab,ti OR 'rectum':ab,ti OR 'sigmoid':ab,ti) AND ('neoplas*':ab,ti OR 'cancer*':ab,ti OR 'carcinoma*':ab,ti OR 'tumour':ab,ti OR 'tumours':ab,ti OR 'metasta*':ab,ti OR 'malig*':ab,ti) | 519967 |
| #4 | #2 OR #3 | 641196 |
| #5 | 'liver tumor'/exp | 364856 |
| #6 | ('liver':ab,ti OR 'hepatic*':ab,ti) AND ('neoplas*':ab,ti OR 'cancer*':ab,ti OR 'carcinoma*':ab,ti OR 'tumour':ab,ti OR 'tumours':ab,ti OR 'metasta*':ab,ti OR 'malig*':ab,ti) | 347012 |
| #7 | #5 OR #6 | 517788 |
| #8 | #1 OR (#4 AND #7) | 85282 |
| #9 | 'surgery'/exp | 6110643 |
| #10 | 'resection*':ab,ti OR 'excision*':ab,ti OR 'surgery*':ab,ti | 2409234 |
| #11 | #9 OR #10 | 6607084 |
| #12 | 'cancer recurrence'/exp | 290132 |
| #13 | 'recurrence*':ab,ti OR 'recrudescence*':ab,ti OR 'relapse*':ab,ti | 899372 |
| #14 | 'earl*':ab,ti | 2898415 |
| #15 | (#12 OR #13) AND #14 | 153391 |
| #16 | #8 AND #11 AND #15 | 1403 |

**Cochrane**

| ID | Search | Hits |
| --- | --- | --- |
| #1 | MeSH descriptor: [Colorectal Neoplasms] explode all trees | 11121 |
| #2 | (colorectal*):ti,ab,kw OR (colon*):ti,ab,kw OR (rectal*):ti,ab,kw OR (rectum):ti,ab,kw OR (sigmoid):ti,ab,kw (Word variations have been searched) | 65528 |
| #3 | (neoplas*):ti,ab,kw OR (cancer*):ti,ab,kw OR (carcinoma*):ti,ab,kw OR (tumour):ti,ab,kw OR (tumours):ti,ab,kw OR (metasta*):ti,ab,kw OR (malig*):ti,ab,kw | 264201 |
| #4 | #1 OR (#2 AND #3) | 33154 |
| #5 | MeSH descriptor: [Liver Neoplasms] explode all trees | 3910 |
| #6 | (liver):ti,ab,kw OR (hepatic*):ti,ab,kw (Word variations have been searched) | 70250 |
| #7 | #5 OR (#3 AND #6) | 22126 |
| #8 | MeSH descriptor: [Surgical Procedures, Operative] explode all trees | 165649 |
| #9 | (resection*):ti,ab,kw OR (excision*):ti,ab,kw OR (surgery*):ti,ab,kw (Word variations have been searched) | 275809 |
| #10 | #8 OR #9 | 342903 |
| #11 | MeSH descriptor: [Recurrence] explode all trees | 14561 |
| #12 | (recurrence*):ti,ab,kw OR (recrudescence*):ti,ab,kw OR (relapse*):ti,ab,kw (Word variations have been searched) | 89942 |
| #13 | (earl*):ti,ab,kw (Word variations have been searched) | 163126 |
| #14 | (#11 OR #12) AND #13 | 13780 |
| #15 | #4 AND #7 AND #10 AND #14 | 70 |

**Web of science**

| # | Query | Results |
| --- | --- | --- |
| 1 | TS=((Colorectal Neoplasms) OR ((colorectal* OR colon* OR rectal* OR rectum OR sigmoid) AND (neoplas* OR cancer* OR carcinoma* OR tumour OR tumours OR metasta* OR malig*))) | 793934 |
| 2 | TS=((Liver Neoplasms) OR ((liver OR hepatic*) AND (neoplas* OR cancer* OR carcinoma* OR tumour OR tumours OR metasta* OR malig*))) | 646220 |
| 3 | TS=((Surgical Procedures, Operative) OR (resection* OR excision* OR surgery*) ) | 4497935 |
| 4 | TS=((recurrence* OR recrudescence* OR relapse*) AND earl*) | 131245 |
| 5 | #1 AND #2 AND #3 AND #4 | 1064 |
