# Supplementary figures and images for: Prognostic factors associated with early recurrence following liver resection for colorectal liver metastases: a systematic review and meta-analysis
Source: BMC Cancer. 2024 Apr 8;24:426. doi: 10.1186/s12885-024-12162-4 (PMC11000331; doi:10.1186/s12885-024-12162-4)

Figure S1. The combination of ER in 21 studies.


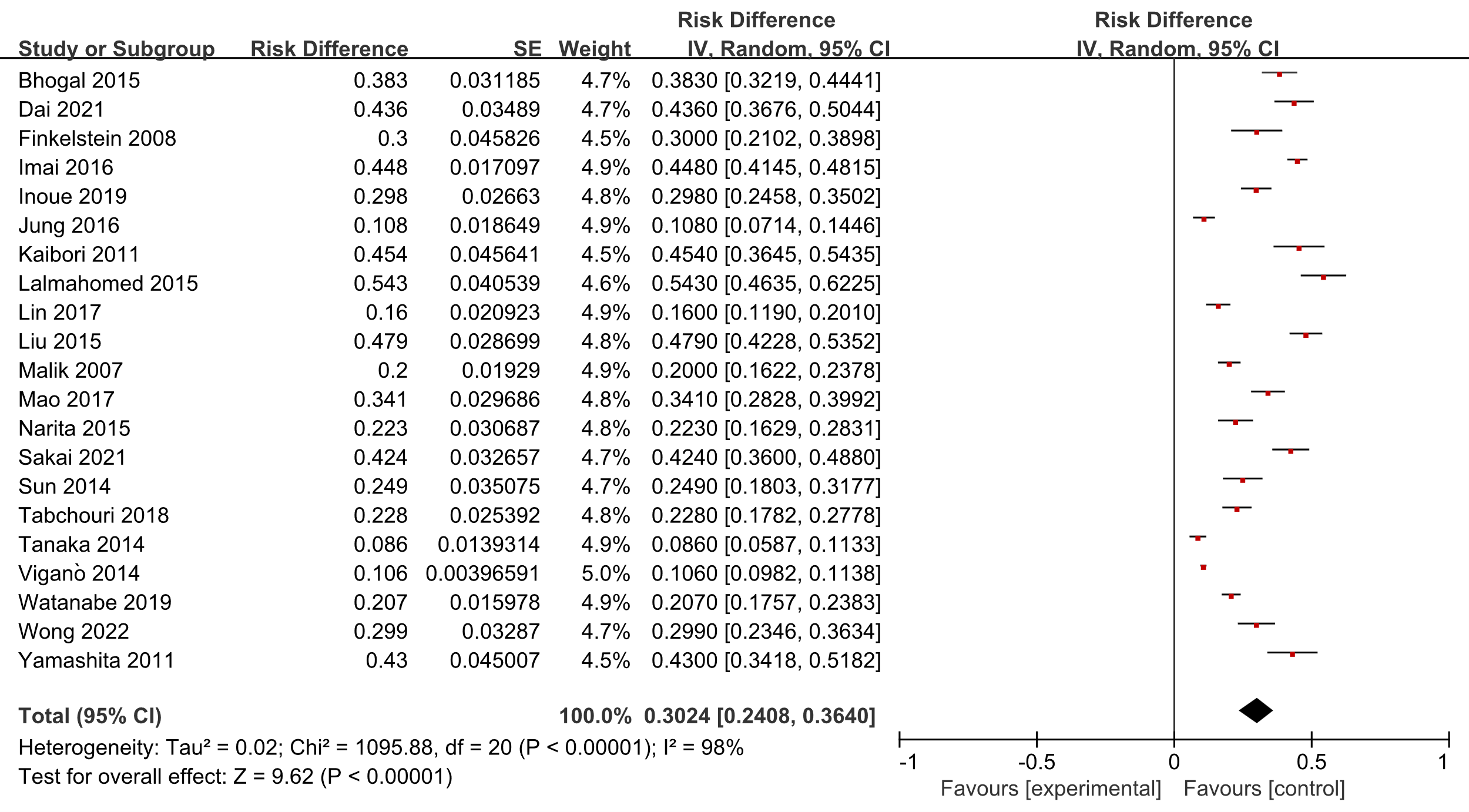

Supplement: Supplementary file 3 — Supplementary Material 3. [file 12885_2024_12162_MOESM3_ESM.docx]

Figure S2. Forest plots of CRS from studies reporting ER after LR for CRLM.


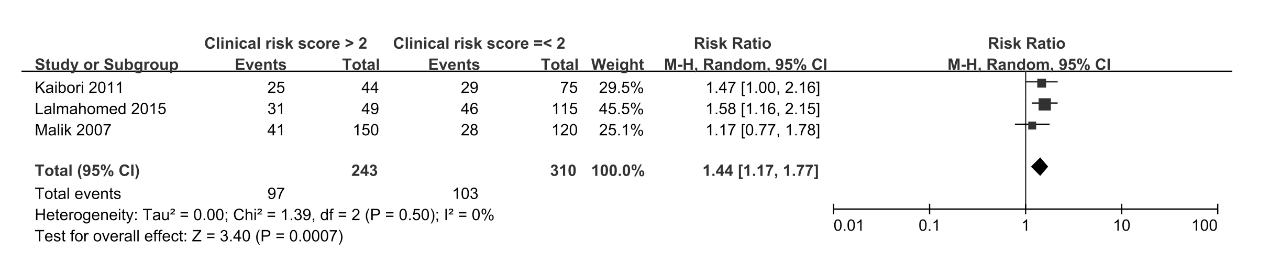

Supplement: Supplementary file 4 — Supplementary Material 4. [file 12885_2024_12162_MOESM4_ESM.docx]

Figure S3. Forest plots of “preoperative chemotherapy” by fixed-effects model.


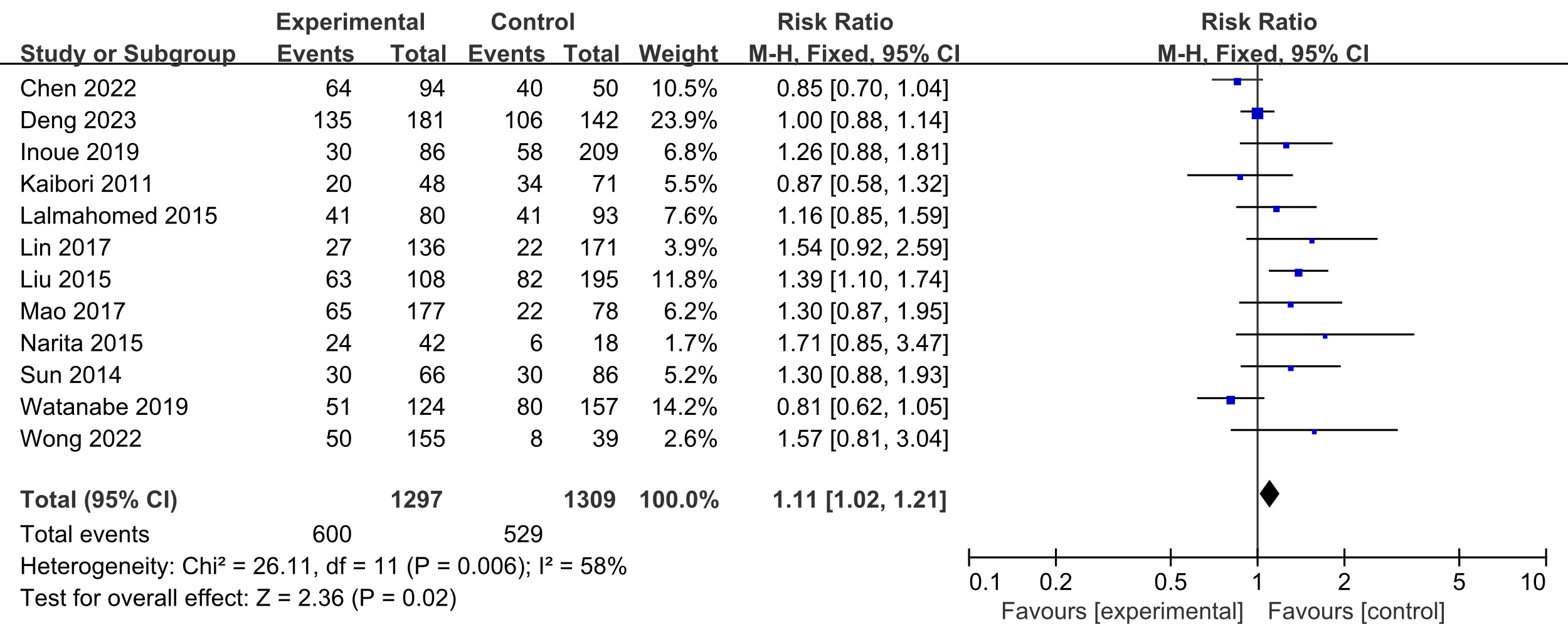

Supplement: Supplementary file 5 — Supplementary Material 5. [file 12885_2024_12162_MOESM5_ESM.docx]
